# A life-threatening case of pregnancy-related atypical Haemolytic uremic syndrome and successful treatment with Eculizumab

**DOI:** 10.1186/s12882-020-02100-4

**Published:** 2020-11-17

**Authors:** Prianka Puri, Anida Hanxhiu, Daniel V. O’Hara, Danny Hsu, Mirna Vucak-Dzumhur

**Affiliations:** 1 The Westmead Hospital Nephrology and Transplant Unit, Westmead, NSW Australia; 2 The Canberra Hospital Nephrology Unit Cabrera ACT, Canberra, Australia; 3 The George Institute for Global Health, Sydney, NSW Australia; 4 The Liverpool Hospital haematology unit, Liverpool, NSW Australia; 5 Western Sydney University and University Notre Dame, Penrith, Australia

**Keywords:** Pregnancy-related atypical Haemolytic uremic syndrome, Eculizumab, Microangiopathic haemolytic anaemia, Thrombotic microangiopathy

## Abstract

**Background:**

Pregnancy-related Atypical Haemolytic Uremic Syndrome (P-aHUS) is a rare condition affecting genetically predisposed women during pregnancy. It is often difficult to diagnose and has a significant impact on maternal and foetal outcomes. It is characterised by microangiopathic haemolytic anaemia and kidney injury from thrombotic microangiopathy.

**Case presentation:**

A 27-year-old female of Lebanese descent presented at 36 weeks’ gestation with foetal death in-utero (FDIU) with placental abruption on a background of previously normal antenatal visits. She was coagulopathic and anaemic with anuric acute kidney injury, requiring emergency Caesarean section, intubation and dialysis. Her coagulopathy rapidly resolved, however, her anaemia and renal dysfunction persisted. A diagnosis of P-aHUS was made, and she was empirically treated with Eculizumab. Her ADAMTS13 level was normal, effectively excluding thrombotic thrombocytopenic purpura. Within 2 weeks of treatment her haematological parameters improved, and her renal function began to recover and within 2 months she became dialysis independent.

**Conclusion:**

This case highlights the challenges of a timely diagnosis of P-aHUS from other pregnancy-related diseases. Although our patient is dialysis-independent, her risk of relapse remains high with subsequent pregnancies. Currently we are awaiting her genetic sequencing to complete her assessment for underlying mutations and are determining the safest approach to a future planned pregnancy.

## Background

During pregnancy, there are constant adaptations within the maternal system to promote the development of the foetus [1]. Part of these adaptations occur at the maternal-foetal interface, whereby there is an increased expression of complement regulator proteins. At the time of delivery, these regulator proteins may be diminished, and an exaggerated maternal response may trigger the excessive activation of the complement system and the development of Pregnancy-related Atypical Haemolytic Uremic Syndrome (P-aHUS). P-aHUS is thought to occur in genetically predisposed individuals who harbour mutations in complement regulator proteins or develop autoantibodies to complement factors. These mutations occur in the alternative complement pathway, most commonly in complement factor H, Complement factor I or mutations in membrane-cofactor protein (MCP) [2]. These proteins inhibit the overactivation of C3 convertase on endothelial surfaces and inhibit the complement cascade. Less commonly, mutations in C3 or complement factor B may be present, which amplify the complement response and confer a poorer prognosis [2].

**Fig. 1:**
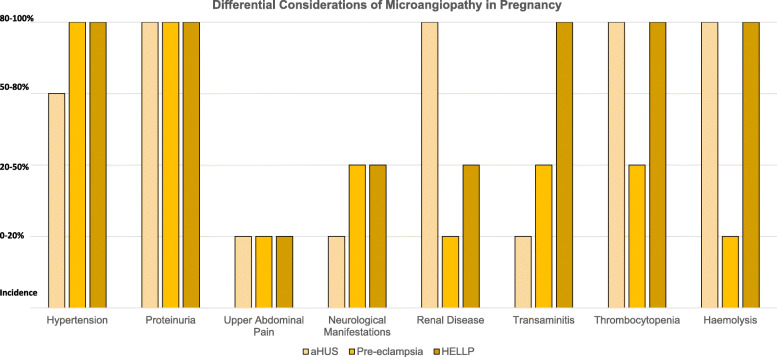
Differential Diagnosis Considerations of Microangiopathy in Pregnancy. Adapted from Bergmann & Rath (2015) [8]

P-aHUS occurs in approximately one in every 25,000 pregnancies and has significant consequences on long-term patient mortality and morbidity [1]. There are disparities between studies as to whether a first or second pregnancy confers the greater risk of developing P-aHUS [3]. A majority of cases occur in the post-partum period [3]. In women with a past medical history of P-aHUS, the risk of recurrence during pregnancy is 20% [4]. P-aHUS involves the presence of microangiopathic haemolytic anaemia (MAHA), thrombocytopenia and acute kidney injury (AKI) from a thrombotic microangiopathy (TMA) [5]. P-aHUS is associated with high rates of end stage renal failure (ESRF) of approximately 78% at 24 months’ post-partum without Eculizumab therapy [6]. This is supported in a retrospective analysis of the aHUS registry including the United Kingdom, France and Italy, which investigated 8-seven patients with P-aHUS [7]. Approximately 70% became dialysis-dependent in the acute setting [7]. At a mean follow up of 7 years later, half had ESRF, one-fifth developed chronic kidney disease and one-fourth required a renal transplant. In the renal transplant group, more than half had recurrence of their aHUS. Diagnosis of P-aHUS requires consideration of the patient presentation and exclusion of similar conditions such as haemolysis, elevated liver enzymes, low platelets (HELLP) syndrome and pre-eclampsia. Patients typically have a greater degree of renal dysfunction with P-aHUS compared to other postpartum cases of MAHA and AKI (Fig. 1).

Preliminary studies suggest Eculizumab therapy reduces the risk of complications associated with P-aHUS, however, its safety during the pregnancy requires serious consideration [4, 5] . Most of the safety data in pregnancy and Eculizumab use is from patients with paroxysmal nocturnal haemoglobinuria (PNH), and to date it has been safely used in these patients, with a minor increased risk of foetal and maternal infection [9]. The majority of data for the use of Eculizumab in aHUS comes from limited case series, which are discussed in further depth below [9–12].

In this case study, we present a case of a young female with placental abruption, triggering a P-aHUS. She has had successful treatment with Eculizumab and is making renal recovery. We await the results of her genetic sequencing and immunotyping to assess for any risk-conferring mutations, while exploring options for our patient’s plans for future pregnancy.

## Case presentation

A 27-year-old female of Lebanese descent presented at 36 weeks’ gestation with abdominal pain and per-vaginal (PV) bleeding. Despite previously unremarkable antenatal reviews, she had a FDIU with placental abruption. This was on a background of a single previous miscarriage at 6 weeks’ gestation with an otherwise unremarkable personal or family history for any other systemic illnesses. On admission she was normotensive, however investigations demonstrated mild kidney injury, anaemia, thrombocytopenia and mild transaminitis (Table 1).

**Table 1:** Trend of patient’s pathology investigations over the course of admission

Investigation	Pre-Admission	Admission	D1	D2	1 week	4 weeks	8–12 weeks	16 weeks
Hb (g/L) (115–165)	148	65	68	78	66	88	114	110
Platelets (150–400 × 10^9/L)	218	Immeasurable	35	26	68	225	259	234
LDH (120–250 UL)	–	2052	2452	2271	1683	249	250	207
Fibrinogen (1.4–4.4 g/L)	–	1.0	2.7	5.8	2.8		–	–
Reticulocytes (50-100 × 10^9/L)	–	188	–	150	93	81	–	–
Haptoglobin (0.3–2.15 g/L)	–	0.19	< 0.15	< 0.15	0.48	1.04	0.78	0.94
Creatinine (45–90 mmol/L)	30	166	213	140	419	326	148	136
eGFR (> 90 ml/min 1.73m^2^)	90	36	27	44	12	16	41	46

**Fig. 2:**
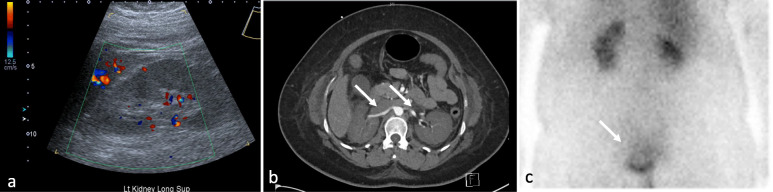
**a**-**c** Renal imaging. Left to right. 2a Ultrasound: Bilateral poor parenchymal perfusion, and poor visualisation of renal arteries and veins. 2b CT angiogram: patent renal arteries with bilateral poor parenchymal perfusion. Renal veins appear patent. 2c MAG3 scan: while markedly reduced renal excretion, there is evidence of some small degree of residual function as demonstrated by tracer excretion into the urinary bladder

She underwent an emergency Caesarean section, was intubated and received multiple blood product transfusions in the setting of coagulopathy and blood loss. She had anuric renal failure and was commenced on haemodialysis. Renal ultrasonography and computed tomography (CT) revealed bilateral poor parenchymal perfusion with patent renal vasculature and mercaptoacetyltriglycine imaging (MAG3) imaging demonstrated evidence of a small degree of residual kidney function (Fig. 2a-c). A renal biopsy was not performed as the patient remained intubated and it was expected that the biopsy would show non-specific thrombotic microangiopathy and would be unlikely to change management. Post-operatively her coagulopathy rapidly resolved with blood product administration, however, her anuric renal failure and MAHA persisted. It remained unclear if she had thrombotic thrombocytopenia (TTP), however, empiric plasma exchange (PLEX) was not performed as her degree of renal injury was not in keeping with TTP and more likely to be related to an alternative cause. Her ADAMTS13 level was nonetheless sent for testing and her direct antigolbuin testing (DAT) was negative (Table 2). Pre-eclampsia with HELLP syndrome was also being considered but felt to be less likely cause.

**Table 2:** Patient’s autoimmune panel results

Investigation	Result
***Autoimmune Screen***	
**Complement 3**	0.76 g/L (0.74–1.57) repeated 12 h later 0.62 g/L
**Complement 4**	0.08 g/L (0.13–0.41) initial, repeated after 2 days normal 0.17 g/L
**Complement 9**	500 mg/L (125–165)
**Complement 5–8**	Normal range
**Complement Factor H antibody**	< 8 (< 100 AU)
**CD46**	1.27 (1.75–4.65 MFI) Crossed checked twice with a health control
**Complement Factor H**	581 mg/L (345–590 mg/L)
**Complement Factor B**	450 mg/L (normal)
**Anti-DsDNA Antibodies**	Negative
**Anti-Nuclear Antibodies**	
**RNP Antibodies**	
**Lupus Anticoagulant**	
**B2-Glycoprotein**	
**Anti-Smith Antibodies**	
**SSA and SSB Antibodies**	
**Ro 52 Antibodies**	
**Scleroderma Ab**	
**Jo-1 Antibodies**	
***Haemolytic Screen***	
**Reticulocytes**	150 × 10^9/L (50–100 × 10^9/L)
**Haptoglobin**	< 0.15 (0.3–2.5 g/L)
**ADAMTS13 Activity**	70% (40–130%; normal)
**Blood Film**	Red cell fragments (1 per high power field). Mild polychromasia. Toxic granulation of neutrophils. Moderate thrombocytopenia.
**Direct Antiglobulin Test**	Negative
**Methylenetetrahydrofolate reductase protein**	Negative gene analysis
**Factor V Leiden mutation**	Not Detected
**The Prothrombin gene mutation**	Not Detected
***Coagulopathy***	
**INR**	1.8 (< 1) Corrected with mixing study
**PT**	19 s (11–18 s) Corrected with mixing study
**APTT**	56 s (24–38 s) Corrected with mixing study
**Protein C Activity**	52% (70–180%)
**Protein S Activity**	39% (55–190%)
***Infection Screen***	
**Toxoplasmosis**	IgM and IgG Negative
**CMV and Parvovirus**	IgG positive. IgM negative.
**Hepatitis B and C**	Negative HCV core antibody and RNA/ Immune HBV surface antibody positive antigens negative

A presumptive diagnosis of P-aHUS was made 4 days after her presentation in the setting of worsening MAHA, resolved coagulopathy, normal blood pressure, negative Shiga-toxin testing and severe renal impairment. Her presentation, in association with FDIU, was in keeping with postpartum risk of P-aHUS and it was expected that HELLP syndrome would have shown signs of improvement by this point. As a result, she was given the quadrivalent and serogroup B Meningococcal vaccinations along with oral Amoxicillin and managed empirically with Eculizumab (900 mg) therapy while remaining dialysis dependent. Five days following her admission, her ADAMTS13 level returned with a normal result (Table 2), effectively excluding TTP. In the days following, her transfusion support was ceased, and she began to produce small amounts of urine by day ten of admission. She was then transitioned to intermittent haemodialysis and a tunnelled internal jugular catheter was inserted. Immunological testing showed abnormally low CD46/MCP level, persistently low C3, and an elevated C9 level in keeping with alternative complement pathway dysregulation. The remainder of her autoimmune screening was negative (Table 2).

Within 2 months of discharge from hospital, her renal function had improved and she became dialysis independent (Table 1). Presently, she remains on fortnightly Eculizumab (1200 mg) and remains in disease remission with normal haematological parameters and stable renal function with a latest eGFR of 60 mL/min/1.73m^2^. In order to predict her risk of relapse and identify a disease mechanism, the patient has undergone flow-cytometric immunophenotyping and whole exome sequencing via the Westmead Hospitals clinical genetics service. Currently, we are awaiting the outcome of any risk-conferring mutations in this patient to stratify her relapse risk.

## Discussion and conclusion

This case highlights the challenges in diagnosis and treatment of P-aHUS in the post-partum period. We believe our patients placental abruption was the inciting event, given her normal haematological and renal parameters during her pregnancy. Our patient received treatment within 4 days, while European data suggests a mean 17-days to diagnosis of the condition [11]. Presently it remains unclear what the optimal timeframe is for the patient to reconsider pregnancy, the appropriate duration of Eculizumab therapy, and the long-term impact on her renal function.

Evidence demonstrates improvement in renal and haematological outcomes following use of Eculizumab therapy in managing P-aHUS [12]. A European retrospective analysis of the aHUS registry demonstrated that out of the four patients that received Eculizumab, three had complete recovery, with the fourth developing ESRF [7] . PLEX was trialled in 78% of the 87 patients in the register, and the risk of ESRF remained 53% with or without this therapy [7]. The Eculizumab results were similar to a Spanish retrospective analysis in which 90% patients who received Eculizumab had renal recovery [11]. However, the appropriate duration of therapy post-diagnosis remains unclear.

The benefits of Eculizumab therapy appear to outweigh its associated risks of infection, with only two cases of severe infection in the postpartum period and no cases of newborn sepsis in a study investigating 75 pregnancies managed with Eculizumab for PNH [9].

Currently, there is no up-to-date protocol regarding Eculizumab therapy in pregnancy, with only several case reports detailing therapy regimens in P-aHUS. Consequently, there are inconsistencies with the dose and duration of Eculizumab used among different cases. In a Spanish Registry Study, therapy was stopped in seven out of the 10 patients after a median duration of 10 months [11] . There was disease recurrence in approximately three of these patients at an average of 6 months post-cessation of therapy, with these patients needing recommencement of therapy [11]. Currently in Australia, therapy is continued for 2 years from the time of commencement, if there is no disease recurrence during this time [13] . More case and prospective data in pregnancy is required to assess optimal timing.

In the case of our patient, it remains unclear when it is safe to consider another pregnancy. Given her renal function has not returned to baseline, we would consider a renal biopsy to prognosticate the degree of chronic renal injury from her episode of P-aHUS, in light of the risk of further deterioration in subsequent pregnancies. Consideration of pregnancy while off therapy heightens the risk of relapse. Current Australian Pharmaceutical benefits scheme (PBS) guidelines approve for 24 months of therapy [13], therefore, pregnancy during this treatment period would be considered as safer. A French retrospective study investigated four patients on Eculizumab therapy with a history of aHUS during their previous pregnancies [11]. The duration from the first diagnosis to conception ranged from six to 18 months [10]. The levels of CH50 were monitored throughout the pregnancy and the maintenance dose of Eculizumab was altered accordingly, with an additional dose also given during labour [10]. While there was no occurrence of aHUS, the incidence of pre-term birth, growth retardation and pre-eclampsia was higher in patients with p-aHUS compared to population average.

The prevalence of complement regulation abnormalities in p-aHUS is reported in 70–86% of cases [14]. This is higher than rates in non-pregnancy related aHUS in which complement abnormalities are reported being up to 50–60% [14, 15]. Most frequent causative mutations in the adult population are related to complement regulator proteins, of note Complement Factor H (CFH), Membrane cofactor protein (MCP, CD46) and Complement factor I (CFI). CFH controls C3 inactivation in the presences of MCP and CFI [15]. CFH mutations are most frequent in up to 20% of all adult cases, followed by mutations in MCP and CFI at 12 and 15% respectively [6, 16]. Loss of function in these regulator proteins results in the inappropriate activation of the alterative complement pathway [6, 10]. Autoantibodies directed at these proteins are more commonly found in the paediatric population [15].

The case of our patient, plasma MCP levels were below refences range compared to healthy controls and there was a decline in serum C3 levels (Table 2). These plasma complement levels can be abnormal in all forms of complement mediated aHUS, and can not infer a causative mutation [4, 16]. Currently we are awaiting the results of our patient’s exome sequencing, the utility of which will allow us to predict her risk of relapse.

Observational data suggests that rate of relapse of aHUS remains high in transplant recipients [14, 15]. Patients with underlying mutations including CFH, MCP and CFI having rate of up to 90, 20 and 80% of disease reoccurrence respectively [6, 15]. Mutations in complement factor B (CFB) have shown to have the highest relapse rate of a 100%, however frequency of CFB mutation remains low at 2% [14, 15]. The use of Eculizumab in p-aHUS has shown to reduce the incidence of relapse in observational studies [4, 5, 9, 10] and therefore being able to predict the risk of diseases reoccurrence based on punitive mutations is imperative in terms of our patients and optimal timing for repeat pregnancy [8, 11].

This case highlights the diagnostic and therapeutic challenges that exist with P-aHUS. We were able to make a prompt diagnosis from the time of presentation and administer empiric therapy. Presently, the patient’s renal function has not returned to baseline, however, her disease appears to be in remission. It remains a challenge to balance the patient’s desire for a future pregnancy, and the optimal timing of this attempt, with her risk of recurrence. Her genetic sequencing may provide insight into her risk of relapse; however, further data is required to assess the safety and efficacy of Eculizumab in the prevention and management of P-aHUS in subsequent pregnancies.

## Data Availability

Data sharing is not applicable to this article as no datasets were generated or analysed during the current study.

## Abbreviations

P-aHUS: Pregnancy-related atypical haemolytic uremic syndrome
FDIU: Foetal death in-utero
MCP: Membrane-cofactor protein
MAHA: Microangiopathic haemolytic anaemia
AKI: Acute kidney injury
TMA: Thrombotic microangiopathy
PNH: Paroxysmal nocturnal haemoglobinuria
ESRF: End stage renal failure
CT: Computed tomography
MAG3: Mercaptoacetyltriglycine imaging
TTP: Thrombotic thrombocytopenia
DAT: Direct antigolbuin testing
PV: Per-vaginal
PLEX: Plasma exchange
